# Three-Dimensional Curved Workflow-Based Optical Coherence Tomography Angiography for Enhancing Atopic Dermatitis Theranostics

**DOI:** 10.34133/research.0778

**Published:** 2025-08-06

**Authors:** Junwei Li, Yunrui Zhang, Ying Huang, Ronghui Li, Kun Wang, Dongbei Guo, Zicheng Huang, Youliang Yao, Yunxin Xue, Guibo Sun, Cheng Jiang, Leyun Wang, Chenzhong Li, Qingliang Zhao

**Affiliations:** ^1^ State Key Laboratory of Vaccines for Infectious Diseases, Xiang An Biomedicine Laboratory, Center for Molecular Imaging and Translational Medicine, Department of Medical Oncology, Xiang’an Hospital of Xiamen University, School of Medicine, School of Public Health, Xiamen University, Xiamen 361102, China.; ^2^ Department of Reproductive Center, Department of Gynecology and Obstetrics, The Ninth Medical Center of PLA General Hospital, Anxiang North Lane, Beijing 100026, China.; ^3^ Key Laboratory of Optoelectronic Science and Technology for Medicine of Ministry of Education, Fujian Normal University, Fuzhou 350117, China.; ^4^ Institute of Medicinal Plant Development, Peking Union Medical College and Chinese Academy of Medical Sciences, Key Laboratory of New Drug Discovery Based on Classic Chinese Medicine Prescription, Chinese Academy of Medical Sciences, Beijing 100193, China.; ^5^ School of Medicine, The Chinese University of Hong Kong, Shenzhen 518172, China.; ^6^ Xiamen Cardiovascular Hospital of Xiamen University, State Key Laboratory of Cellular Stress Biology, Fujian Provincial Key Laboratory of Reproductive Health Research, Department of Obstetrics and Gynecology, School of Medicine, Xiamen University, Xiamen 361102, China.

## Abstract

Optical coherence tomography angiography (OCTA) is a major advancement in imaging, offering high-resolution microvascular volumetric images crucial for diagnosing and studying dermatological diseases. However, current data analysis and clinical evaluation criteria primarily rely on 2-dimensional (2D) imaging results, resulting in imprecise diagnoses due to the substantial loss of 3D curved structures and microvascular details. To address this issue, we propose a high-fidelity 3D curved processing workflow that integrates an artificial neural network (ANN) with a 3D denoising algorithm based on the curvelet transform and optimal orientation flow (OOF). This innovative workflow enables precise 3D segmentation and accurate quantification of dermal layer microvasculature in atopic dermatitis (AD) in vivo. Furthermore, the use of 3D multiparametric microvasculature quantitative metrics establishes a robust framework for assessing the efficacy of AD treatments in 3D images. Our study results demonstrate that skin structure imaging and the dynamic evolution of 3D microvasculature align with observed pathological changes. Compared to traditional 2D analysis, the maximum variation rate of 3D curved multiparametric information is approximately 10%. Consequently, our research marks a significant advancement in the accurate quantification of microvasculature in AD development and theranostics, paving the way for the clinical application of OCTA in dermatology.

## Introduction

Atopic dermatitis (AD) is the most common chronic, inflammatory disorder characterized by intense pruritus, erythema, dry, and a disrupted epidermal barrier protection [1,2]. Studies estimate that approximately 15 to 20% of children and 2 to 10% of adults worldwide suffer from this condition, with incidence rates increasing globally [3]. Beyond its impact on skin integrity, AD poses serious health risks, including secondary bacterial and viral infections because of a compromised skin barrier, as well as an increased likelihood of developing systemic inflammatory conditions. Additionally, its persistent pruritus and visible lesions contribute to substantial psychological distress, leading to sleep disturbances, anxiety, and depression, which further impact patients’ quality of life [4,5].

Current clinical imaging evaluation of atopic AD primarily relies on tools such as dermoscopy, confocal laser scanning microscopy (CLSM), high-frequency ultrasound (HFUS), and microcomputed tomography to enhance diagnostic accuracy and treatment monitoring [6,7]. While these techniques provide valuable insights into AD severity, they are often limited in assessing microstructure and vascular changes in vivo at high resolution. Emerging methods like laser Doppler perfusion imaging (LDPI) and laser speckle contrast imaging (LSCI) are useful for routine assessments but have notable limitations in evaluating vascular changes and treatment response in AD [8]. LDPI’s low spatial resolution restricts its ability to analyze microvasculature and quantify vascular density, while LSCI, although effective for real-time hemodynamic monitoring, is sensitive to motion artifacts and lacks 3-dimensional (3D) vascular structural information [9]. Accordingly, to address these limitations, developing a robust and objective evaluation method based on image microstructure and vessel-based scoring is essential for providing reliable guidance in preclinical decision-making, optimizing treatment regimens, and assessing long-term treatment responses.

Optical coherence tomography (OCT) is a promising, noninvasive, and label-free imaging modality that utilizes low-coherence interferometry to provide 3D structural image with micrometer-level resolution in both longitudinal and lateral dimensions [10,11]. Based on this imaging mechanism, OCT enables real-time imaging without the need for biopsies or contrast agents, provides clear distinction between different skin layers, and allows visualization of structures beyond the reach of conventional dermoscopy [12–14]. Thus, it is widely used in diagnosing various dermatological conditions, including cancers, inflammatory diseases, and infections [13–16]. Building on the OCT technique, OCT angiography (OCTA) was developed to generate volumetric angiographic images using the same principle of low-coherence interferometry to capture backscattered light from different depths within the tissue. OCTA differentiates between static and dynamic tissue components by analyzing temporal changes in OCT signal intensity or phase [17–20]. Due to decorrelation, stationary objects such as tissue and air are suppressed, while moving objects, such as blood flow, are enhanced. In general, both OCT and OCTA images can be acquired during an OCTA examination, allowing for the simultaneous visualization of structural and vascular information. OCTA provides 3D volumetric data, enabling the segmentation of vascular networks across different tissue layers, such as the epidermis and dermis in skin imaging or the retinal capillary layers in ophthalmology [21–25].

The development of AD is often accompanied by changes in the skin layer structure and blood vessels [26–28]. OCTA has revolutionized the study of AD by providing unparalleled insights into morphological abnormalities of the cutaneous microvasculature, including vascular dilation, increased vessel density, and enhanced microvascular complexity [29–31]. These anomalies are closely linked to the inflammatory milieu and the progressive severity of AD. By precisely quantifying 3D vascular parameters, OCTA not only offers rigorous quantitative data on the cutaneous microvascular landscape in AD patients but also enables dynamic monitoring of disease progression and therapeutic responses [32–34]. Additionally, several deep learning strategies have been reported for performing 3D reconstruction, quantifying vessel parameters and evaluating various angioarchitectural properties [35–39]. However, image artifacts that arise during the image acquisition stage can affect the quality of raw OCT data, leading to issues such as low contrast and noise [40–42]. While these artifacts can be mitigated through proper recognition and reacquisition of data, they may still impact the accuracy of quantitative parameter extraction and the quality of visualization in OCTA [43–46]. In addition, the current studies are limited to the 2D flattened images, which severely lost the abundance of 3D curved microvascular information in the depth direction.

**Fig. 1.**
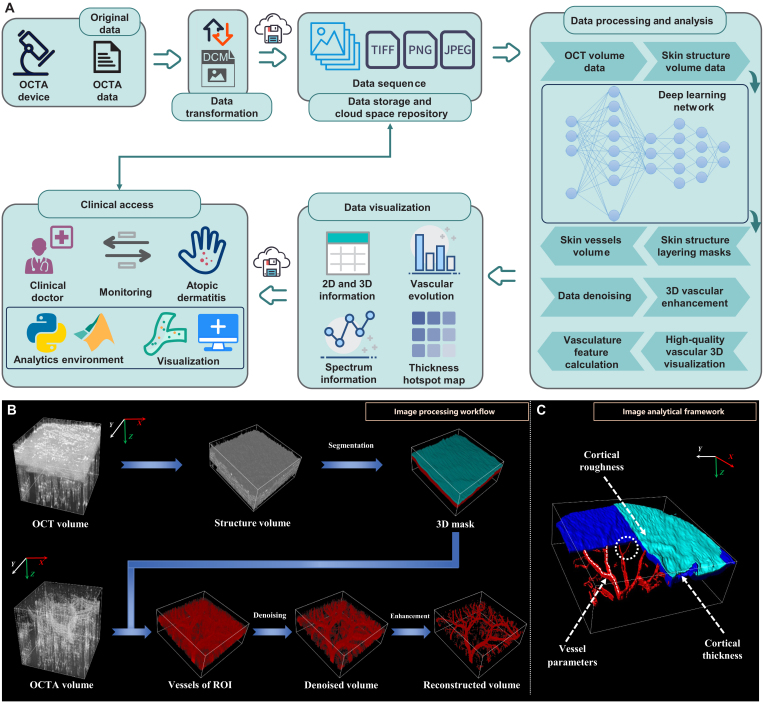
Schematic of the proposed processing and analyzing framework. (A) Detailed experimental flow, data processing, and analysis process. (B) 3D volume processing method in detail. (C) Framework quantitative parameters extracted from the processed 3D volume.

Herein, we propose a novel deep learning-based 3D curved image processing workflow and analysis framework for complete 3D tissue reconstruction and microvascular evaluation (Fig. 1). The image processing workflow aims to generate high-quality 3D image structural and flow volumes without the requirement for registration. The proposed analysis framework is used in 2 aspects to comprehensively examine targets. High-resolution 3D rendered images are displayed. Quantitative analyses are then applied to process volumes, including tissue thickness and various vessel parameters. Furthermore, we have used this framework to monitor AD on mouse to demonstrate its great potential for effective and comprehensive information extraction. Furthermore, compared to 2D results, a higher rate of change indicates that analyzing blood vessels based on a 3D curved transform framework is more sensitive to subtle changes. Our study is the first to demonstrate that the 3D curved workflow method offers sufficient sensitivity and high fidelity to enhance the clinical application of OCTA in dermatologic diseases.

## Results

### Swept-source OCT/OCTA visualizes the microstructure and vasculatures

**Fig. 2.**
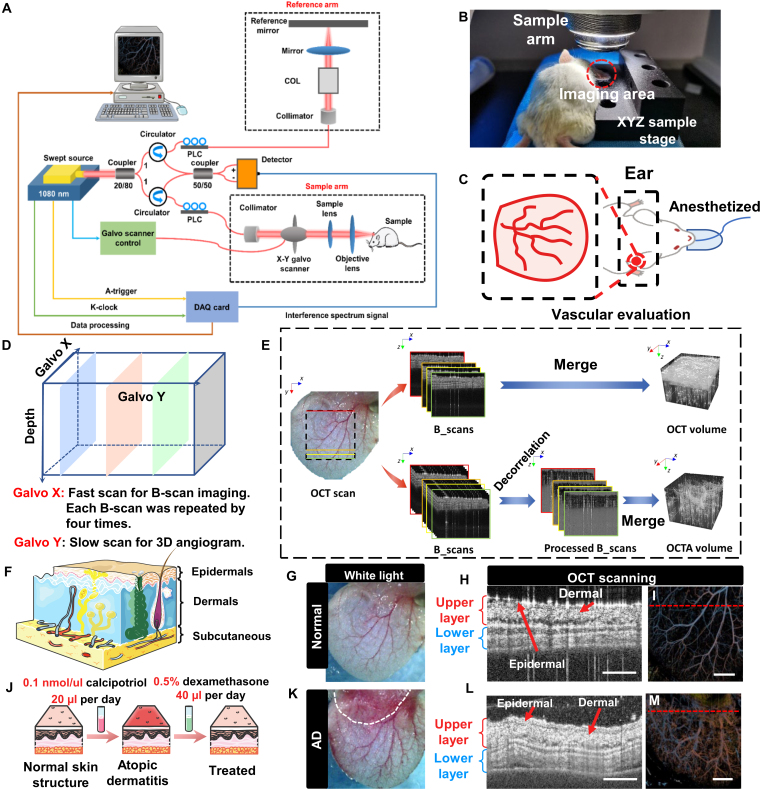
(A) Schematic diagram of OCT system. (B) Photograph of the OCT imaging of the mouse ear. (C) Schematic of imaging of mouse ear. (D) OCT *XY* direction scanning. (E) Different ways of OCT and OCTA on image reconstruction. (F) Skin layer’s structure diagram. (F and J) Skin layer’s structure diagram and modeling and treatment strategies, respectively. (G and K) White light and OCT results of a mouse ear before and after modeling. (H and L) Layer structure of skin before and after AD image as the red dotted line in (I) and (M). Scale bar, 2 mm. (I) and (M) OCT en face image of normal skin and AD, according to (G) and (K), respectively. Scale bar, 2 mm.

Figure 2E presents the OCT and OCTA volumes before and after AD treatment. Figure 2G to I, along with Fig. 2K to M, illustrates the microstructure of 2 distinct skin layers and the microvascular network in normal and AD-affected skin. Notably, Fig. 2I and M highlights key differences between normal and pathological skin. In the white light images, redness and swelling were evident in the ears of mice with AD. OCT B-scans and en face images clearly revealed structural changes, demonstrating that pathological skin was thicker than normal skin and exhibited a more complex vascular network. Additionally, Fig. 2I and M shows a significantly increased density of microvessels in lesional AD compared to healthy skin. This vascular alteration is primarily driven by chronic inflammation, which induces the up-regulation of pro-angiogenic factors such as vascular endothelial growth factor [4,5].

### 3D reconstruction and segmentation of OCT/OCTA image

**Fig. 3.**
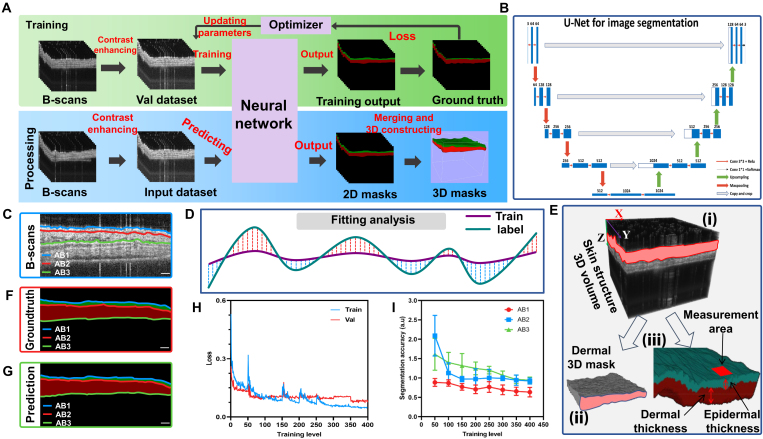
(A) An overview of skin layer segmentation for 3D OCT images. (B) U-net structure. (C, F, and G) Original B-scans, ground truth, and prediction from top to bottom, and 3 border lines are marked in different colors: the background–epidermal interface (blue line, AB1), the epidermal–dermal junction (red line, AB2), and the dermal–background interface (green line, AB3). (D) The bottom indicates a schematic of quantitative data calculation, whereas the below image indicates quantitative data results. (H) Loss value of training level. (I) Accuracy rate of segmentation with training level. (E) 3D skin layer segmentation results, showing the 3D skin structure volume (i), dermal 3D mask (ii), and 3D skin layer structure volume segmented (iii), respectively. Scale bar, 1 mm (C, F, and G).

Figure 3A illustrates the full workflow of volume segmentation, encompassing both the training and processing stages. To evaluate segmentation performance, we compute comparable parameters between the predicted results and the ground truth. Due to the highly unbalanced distribution between the background and cortex regions, pixel matching-based metrics exhibit minimal variation, making it challenging to effectively assess segmentation accuracy. As shown in Fig. 3C, F, and G, 3 key boundaries are delineated: the background–epidermal interface (blue line, AB1), the epidermal–dermal junction (red line, AB2), and the dermal–background interface (green line, AB3). Given that the segmentation area is intrinsically linked to the spatial positioning of these boundaries, assessing their positional variations offers a robust and indirect method for evaluating segmentation performance. The computed results, presented in Fig. 3D, indicate a high degree of segmentation accuracy, aligning closely with the performance observed during the training phase.

### 3D vessel image denoising and enhancement

In practice, tailing artifacts are commonly present in raw 3D OCTA images, and it is seriously affecting the readability of the image. These artifacts occur primarily in the axial direction above blood vessels, particularly large vessels, and manifest as false flow signals with slightly lower intensity than the true vessel signal. Due to their morphological similarity to actual blood vessels, these artifacts can be mistakenly identified as vasculature by certain curve detection algorithms, leading to inaccuracies in vessel segmentation. Consequently, the presence of these false vessels can distort the calculation of vascular parameters, ultimately compromising the accuracy of quantitative analysis.

**Fig. 4.**
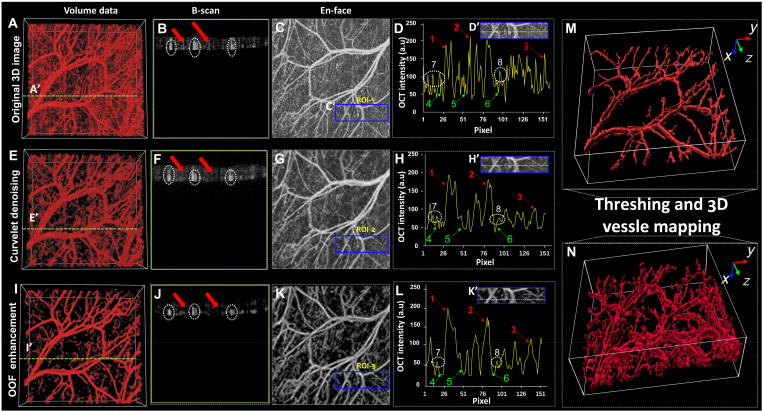
(A) Original 3D OCTA volume. (E) 3D OCTA image after curvelet denoising. (I) 3D OCTA image after OFF vessel enhancement. (B, F, and J) B-scan images along the yellow dotted lines (A′), (E′), and (I′) in (A), (E), and (I), respectively. The white dotted circles indicate blood vessel signals, while the red arrows highlight striped noise areas. (C, G, and K) En face images of the original, curvelet-denoised, and OFF-enhanced 3D OCTA images, respectively. (D, H, and L) 1D signals extracted along the yellow dotted lines in (D′), (H′), and (K′), corresponding to ROI-1, ROI-2, and ROI-3 in (C), (J), and (K). Red arrows (1 to 3) indicate vascular signal areas, green arrows (4 to 6) highlight background signal areas, and white dotted circles (7 and 8) mark regions affected by artifacts. (M and N) Rendered 3D microvascular volumes of normal skin and AD.

To mitigate their impact, a hard thresholding method was used to remove the noise. As shown in Fig. 4A, the original 3D OCTA vessel image after curvelet denoising and OOF processing (Fig. 4I), the intensity of the vessel is slightly reduced, and the background is greatly suppressed. From Fig. 4G, we can observe that the image contrast has improved after curvelet denoising. Additionally, the 1D signals in Fig. 4H indicate that the Gaussian noise has been effectively suppressed compared to Fig. 4D. After 2 filters, the Gaussian and striped noises are simultaneously suppressed in Fig. 4I. Figure 4M and N presents the magnified 3D vessels of normal skin and AD after denoising and filtering, where neovascularization is clearly visible. The blood vessel density in AD, as shown in Fig. 4N, is significantly higher than in Fig. 4M. This result suggests that AD leads to localized increases in microvascular density.

### Extraction of multiparametric blood vessel information

Skin diseases such as AD always develop with changes in blood vessels within the corresponding dermis layer. Artificial interpretation of medical images is insufficient in terms of efficiency and accuracy. Therefore, in addition to visual representation, vascular parameters based on 3D OCTA images are also required to quantitatively reveal vascular changes with disease progression. A comparison with 2D images based on maximum intensity projection (MIP) is also made to demonstrate the potential of computer-aided diagnoses in dermatitis. The analysis of raw data is often compromised by image quality issues, including low contrast and various artifacts, which can hinder the accuracy of results. However, following processing with the proposed workflow, the reconstructed images demonstrate improved suitability for image data mining. Utilizing these enhanced images, we applied the 4 blood vessel parameters described in the previous section to conduct a preliminary investigation of blood vessel structure.

**Fig. 5.**
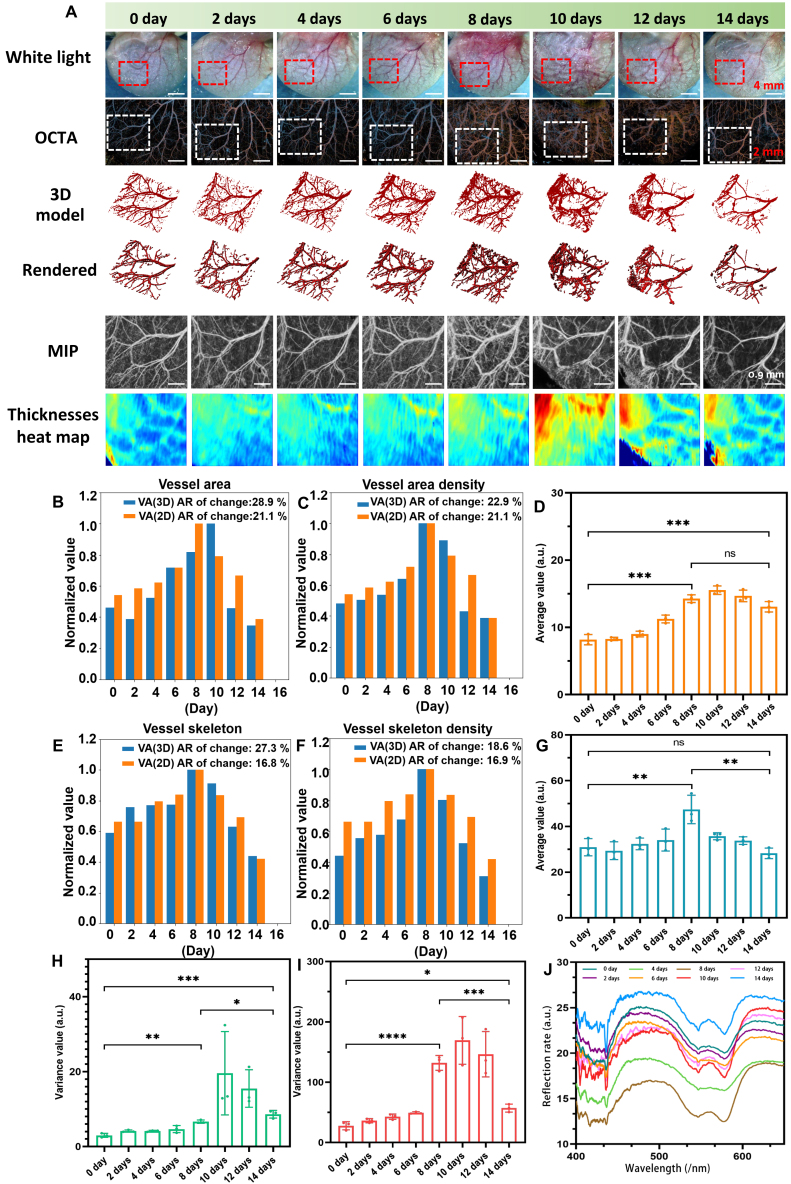
(A) Image data obtained by different processing methods. (B, C, E, and F) Statistical diagram of blood vessel parameters of VA, VAD, VS, and VSD, respectively. (D and G to I) Average value and the variance value of dermal and epidermal layer thickness change. (J) Reflection spectrum of mouse ear at different days. AR, average rate.

Figure 5A shows the image data obtained by different processing methods. We selected a rectangular area of 360 × 360 pixels as the region of interest as shown in Fig. 5A (red dotted box in white light image) from 0 to 14 d. Corresponding OCTA images are also displayed on the second line in Fig. 5A. Meanwhile, the 3D models, 3D rendered, MIP, and thickness heat map results are plotted in Fig. 5A. We can observe that vascular networks become more complex during AD development with the growth of the primary vessels and the emergence of microvasculature. On the other hand, during the first 3 d, when disease modeling was still under development, little variation was observed between white light and 3D images. To further evaluate early changes in AD progression, we extract quantitative data based on OCTA images. The results of blood vessel parameters are shown in Table and plotted in Fig. 5B, C, E, and F; we can see that all 4 parameters increased during AD modeling and decreased afterward. The change rates of the parameters are not less than 10%. More importantly, during the first 3 d of modeling, the changing rate of change is not less than 10%. Quantitative parameters clearly describe the daily process of AD. To make a comparison with volume-based extraction, we also obtained quantitative data of 2D MIP images and drew as shown in Fig. 5B. Because these images are projected from reconstructed blood vessel volumes, they do not require any additional vessel enhancement. Analysis of 2D images yielded nearly the same result, but the changing rate of change was generally lower, suggesting that 3D results may reflect or detect more subtle vessel changes than 2D results.

**Table.** Multiparametric vessel parameters based on 2D and 3D data

		Normal	Modeling				Treating			Average rate of change
Day	Vessel Parameters (VPs)	0	2	4	6	8	10	12	14	
3D	VA	278,504	234,977	317,641	434,710	495,952	605,974	276,343	210,057	0.289
	VS	7,968	10,078	10,451	12,230	18,075	14,495	9,430	5,560	0.273
	VAD	0.0754	0.0790	0.0844	0.1007	0.1569	0.1398	0.0677	0.0604	0.229
	VSD	0.00216	0.00277	0.00282	0.00283	0.00366	0.00334	0.00231	0.00160	0.186
2D	VA	35,553	38,541	41,029	47,283	65,793	52,166	43,737	25,485	0.211
	VSD	6,720	6,721	8,066	8,499	10,134	8,459	7,020	4,248	0.168
	VS	0.2743	0.2974	0.3166	0.3648	0.5077	0.4025	0.3375	0.1966	0.211
	VAD	0.05185	0.05186	0.06224	0.06558	0.07819	0.06527	0.05417	0.03278	0.169

Measurement of thickness is based on segmented structure volume in Fig. 3EIII, and then we counted the average layer thickness, and its variance parameters of the epidermal thickness and dermal thickness are presented in Fig. 5D, G, H, and I. Results show that both the dermis and epidermis significantly become thickened and rough as the AD progresses. In addition, ear thickness is plotted as hot spot map shown in the last row in Fig. 5A as an intuitive demonstration. Reflection spectrum reflects surface uniformity. We also explored the thickness of epidermal and dermal layer and reflection spectrum on ear in Fig. 5J. As Fig. 5J shows, spectrum results proved part of conclusion based on thickness data: AD makes the skin surface rougher.

## Discussion

OCTA offers an unparalleled capability for noninvasive, label-free, and depth-resolved visualization of the vasculature network at single-capillary-level resolution in vivo. However, current studies are largely confined to 2D flattened images, resulting in a substantial loss of microvascular information along the depth dimension. In this work, we present a novel 3D curved deep learning-based image processing workflow, integrating artificial intelligence-based methods for 3D curved tissue structure and blood vessel signal processing. Moreover, to improve the quality of 3D curved volumetric reconstruction and enhance the accuracy of multiparametric quantification, we developed a 3D signal processing method based on the curvelet transform and OOF framework. The efficacy and exceptional fidelity of this innovative framework were validated in live mice with AD before and after treatment. Additionally, the dynamic progression of AD was analyzed by calculating multiparametric morphological changes in the skin layers and blood vessels. Notably, compared to 2D analysis, the 3D curved transform framework demonstrated a higher sensitivity to subtle changes in blood vessels, as reflected in the higher rate of change observed. This approach enables precise segmentation and quantification of 3D curved information within deeper dermal layers.

Additionally, from a clinical perspective, our workflow offers significant instructional value by enabling dermatologists to directly visualize microvascular alterations associated with AD on 3D curved skin surfaces, addressing the limitations of conventional 2D imaging. This enhanced visualization can support the development of personalized treatment strategies, facilitate precise monitoring of disease progression, and improve the evaluation of therapeutic responses. Furthermore, the findings of this study present an opportunity to create training modules for clinicians, focusing on the interpretation of 3D vascular features and their correlation with AD pathology, thereby advancing clinical expertise in diagnosing and managing skin conditions.

Although the effectiveness of the method was validated, this study has several limitations, including a small sample size, a focus on a single disease type, and limited imaging depth. Future studies could address these limitations by including diverse skin disease types, conducting dynamic long-range monitoring on a larger sample size, and integrating histological analyses such as hematoxylin and eosin or Masson’s trichrome staining to assess epidermal thickening, dermal inflammatory cell infiltration, and collagen deposition. Additionally, immunohistochemistry could be employed to detect key inflammatory markers, further enhancing the understanding of AD disease mechanisms.

In summary, we propose a novel 3D curved-based OCTA image processing workflow designed to generate high-quality structural and vascular volumes. This method effectively addresses the challenges of low-definition imaging and loss of detail inherent in conventional OCT scans. To evaluate its utility, we developed an analytical framework leveraging both structural and vascular volumes to monitor the progression of AD in mouse skin. On one hand, the proposed approach enables high-fidelity 3D local vascular reconstruction, offering rapid and intuitive visualization of vessel morphology and architecture. This highlights the remarkable imaging capabilities of OCT in capturing superficial vascular networks. On the other hand, we performed a multiparameter quantitative analysis based on high-resolution 3D vascular volumes, facilitating an in-depth investigation of AD progression. Beyond its skin application in dermatological research, this structured analytical framework demonstrates the potential to significantly expand the clinical utility of OCT. The proposed workflow not only enhances dermatological imaging but also holds substantial promise for broader applications in medical image processing and analysis, particularly in high-precision diagnostics and disease monitoring

## Materials and Methods

### AD model building and experiment

MC903 (calcipotriol, a synthetic analog of vitamin D3) is frequently employed to establish murine models of AD [47,48]. Its mechanism is predominantly characterized by the activation of keratinocytes and immune cells, resulting in the secretion of pro-inflammatory cytokines [e.g., interleukin-6 (IL-6), IL-1β, and tumor necrosis factor-α (TNF-α)] and subsequent induction of localized inflammatory cascades. This model accurately recapitulates the hallmark pathological manifestations of human AD, encompassing cutaneous inflammation, epidermal barrier disruption, and immune cell infiltration [49]. MC903 is obtained from a commercial supplier (Sigma-Aldrich Corp., St. Louis, MO, USA). MC903 was dissolved in anhydrous ethanol or propylene glycol to prepare a 100 μg/ml (10 μg/μl) stock solution. Before each experiment, the stock solution was diluted to a final volume of 20 μl at a concentration of 0.1 nmol/μl, and stored at −20 °C, protected from light. Laboratory mice of the Balb/c strain, approximately 4 to 6 weeks old, were selected, with 6 mice in each group.

Animals were housed in a controlled environment (22 ± 2 °C, 50 ± 10% humidity) with a 12-h light/dark cycle, provided with standard chow and water ad libitum, and monitored daily for health. Acclimatization was allowed for 7 d prior to experiments. They were divided into 2 groups. One day before the experiment, hair was carefully removed from the target area using depilatory cream or small scissors to avoid damaging the skin. The skin was checked to ensure it was clean and intact, and alcohol swabs were used to clean the area if necessary. For ear AD inflammation model, 20 μl of 0.1 nmol/μl MC903 mixed with ethanol solution was applied on the outer surface of each ear every day. The treatment was administered once daily for consecutive days, depending on the desired level of inflammation. A stable temperature (22 to 25 °C) was maintained, and external stress or disturbances for the mice were minimized. Treatment was initiated 4 d later; dexamethasone was applied to the mice in the experimental group, and the same amount of ethanol was applied to the control group.

To minimize breathing and micromovement effects, mice were lightly anesthetized with 2% to 3% isoflurane in oxygen for 2 to 3 min to ensure physiological stability during imaging. Throughout the experiment, they were placed on a heating pad to maintain body temperature, while their limbs were secured with tape to stabilize breathing and reduce micromotion. An *XYZ* 3D adjustment platform was used for precise focusing to achieve optimal image quality. As shown in Fig. 2B, the mouse ear was flattened for white light imaging and OCT examination at the same location. The OCT scans were completed within this period, supported by high-speed scanning to reduce motion artifacts. Residual motion artifacts, such as those from breathing, were corrected using a multiple wavelet- fast Fourier transform (Wavelet-FFT) algorithm during postprocessing, as described in our previous research [41]. All mice were sacrificed at the conclusion of the experiments, and all procedures were conducted in strict accordance with the guidelines of the Xiamen University Animal Care and Use Committee (protocol number: XMULAC20190146).

### OCT system and in vivo imaging

This study was conducted using a homebuilt swept-source OCT system. As shown in Fig. 2A, the system consisted of a swept-source laser (Axsun 105, AXSUN Technologies Inc., Billerica, MA) with a central wavelength of 1,064 nm, operating at a scanning rate of 200 kHz. The OCT signals from the balanced detector were sampled by a high-speed digitizer (400 MHz, 12-bit up to 1.8 GS/s, AlazarTech, ATS9360) using the clock signals and built-in trigger of the Axsun laser. The system achieved an axial resolution of approximately 5 μm in the air and a lateral resolution of approximately 10 μm. During the in vivo experiment, the scanning protocol consisted of 800 × 800 A-lines for 3D OCT structural imaging and 800 × 800 × 4 A-lines for OCTA, covering an approximately 10 × 10 mm^2^ field of view (FOV). A single 3D OCT structural scan required approximately 6 s, while an OCTA scan took about 26 s to complete. More details of the system have been described in our previous study [50]. During the in vivo imaging, the mouse ear was flattened for white light imaging and OCT examination at the same location. The OCT scans were completed within this period, supported by high-speed scanning to reduce motion artifacts.

### 3D reconstruction and segmentation of 3D structural volumes

The OCT examination is performed at approximately the same time of the day. A single check generates 2 raw 3D datasets that store structural and vascular information of the same area. The 2 volumes are then input into the proposed workflow to obtain high-quality 3D reconstruction results. Although we attempt to ensure the same scanning operations. The scanning environment has been subtly changed over time, resulting in different acquisition effects. Therefore, the parameters require slight adjustments in the workflow for different OCTA images to obtain the best quality. Furthermore, we applied the proposed 3D data processing workflow to OCTA images of the mouse ear from modeling to treatment. The complete workflow of volume segmentation, including training and processing steps, is illustrated in Fig. 3A. Then, the 3D curved structural volume is segmented using an artificial neural network (ANN) to produce masks and extract 3D spatial locations of each layered structure (Fig. 3B). Furthermore, we used 3D signal processing based on curvelet transformation and optimally oriented flux (OOF) for denoising the 3D blood flow image. Meanwhile, we selected a global threshold value for blood vessel reconstruction. The output can be further segmented by the masks into vessels in different structural areas, providing a platform for structural analysis of blood vessels (Fig. 3E).

Although 3D data can be directly segmented using ANN, the manual labeling is a time-consuming process and incurs an expensive expense. Therefore, we first cut them into 2D images as the network input. 2D B-scans, which are cut from structural volumes, have low contrast, which makes manual segmentation difficult. We used contrast limited adaptive histogram equalization (CLAHE) to enhance the contrast of B-scans and use them as network input based on U-Net [51]. U-Net is a convolutional neural network (CNN) for image segmentation with U-shaped architecture. It consists of an encoder (contracting path) that extracts features through convolution and pooling layers and a decoder (expanding path) with skip connections to preserve spatial details. This design enables precise pixel-wise segmentation and is one of the most widely used neural network models in biomedical image processing. The architecture of U-Net is illustrated in Fig. 3B and includes contraction and expansion paths. The contraction path consists of two 3 × 3 convolution operations and rectified linear unit (ReLU) operations and a 2 × 2 max pooling operation for down-sampling. This type of structure compresses the image into a dense feature vector with smaller spatial dimensions to extract deeper levels of features. The expansion path includes two 3 × 3 convolution operations and ReLU and one 2 × 2 convolution operation for up-sampling. Before each expansive path, a concatenation is conducted using the corresponding feature map. It decodes the vector from both low- and high-dimensional feature maps to generate a segmented image.

The output of the network is compared with the ground truth to generate a loss using a self-defined calculation algorithm. Subsequently, the loss is then used to update the network parameters for more accurate segmentation performance, resulting in a well-trained segmentation model. Next, the raw data are cropped solely in the transverse direction to create B-scans. All these 2D images are fed into the model to generate 2D masks. These 2D masks are then merged into 3D masks, containing 3D positional information about the dermis layer (Fig. 3A). The output mask is used to calculate skin layer thickness and extract 3D vessels in specific layers [19].

We collected 70 B-scans from different mouse ears during the modeling of the treatment processes, and they were manually segmented as ground truth. The 10-fold cross-validation was applied for data collection partitioning and model evaluation. The training process was implemented on a computer equipped with an Intel 10700H central processing unit, 128 GB memory, and NVIDIA A5000 GPU (16 GB memory). The training and validation loss are shown in Fig. 3D. The performance of the segmentation model, or the image segmentation effect, can be reflected in the image similarity. Therefore, we calculate similar parameters between the prediction and the ground truth. For the highly unbalanced proportion of background and cortex areas, the pixel matching-based parameters exhibit little variation, making it difficult to describe the changes in segmentation effects. As shown in Fig. 3C, F, and G, we extracted 3 borders: background–epidermal, epidermal–dermal, and dermal–background. The segmentation area is closely related to the location of the 3 lines; thus, we can indirectly explore segmentation effect based on the location changes of the lines. Calculation results are shown in Fig. 3D, and segmentation results achieved high level as the training process in Fig. 3H and I.

### Artifact removal of 3D vascular volumes

OCTA images are generated by calculating the decorrelation of repeated B-scans; however, this process also captures noise-related fluctuations in the OCT signals. Two primary types of noise commonly present in raw data are stripe noise, induced by relative motion between the sample and the detector, and salt-and-pepper noise, resulting from the stochastic nature of photon scattering. Figure 4 illustrates the raw OCTA data affected by noise, where 2 red dots indicate stripe artifacts in the B-scan. A graph plotted along the yellow line in the en face view reveals high-frequency signal protuberances caused by striped artifacts and Gaussian noise. Moreover, the inherently low contrast between the vessel signal and the background presents a significant challenge for accurate vessel detection. This limitation contributes to the presence of multiple high-signal regions that contain extraneous information, thereby constraining both the qualitative and quantitative accuracy of the analysis. To address these challenges, we implemented a 2-step approach utilizing a 3D signal processing method, which effectively reduces noise and enhances vascular visualization.

To remove artifacts from the vessel volume, we employ the 3D curvelet transform to separate the noise signal from the background while preserving as much of the vessel signal as possible. Unlike the wavelet transform, 3D curvelets are localized not only in the space–frequency domain but also across multiple orientations, enhancing directional selectivity. This property enables more effective noise suppression while maintaining the structural integrity of vascular features. Therefore, the scale and orientation characteristics of an image can be separated into different sub-bands using the curvelet transform. Images in curvelet domain can be referred as:

$$C_{s,w,k1,k2,k3}\;=\sum_{0\leq x<M,0\leq y<N,0\leq z<L}f(x,y,z)*\Phi_{s,w,k1,k2,k3}[x,y,z]$$ (1)

where f (*x, y, z*) refers to the 3D volume in the spatial domain; *s* and *w* refer to scale and direction coefficients, respectively; *k1*, *k2*, and *k3* are the location factors. Gaussian noise is distributed throughout the spatial domain, and its intensity is generally lower than that of the foreground information. Therefore, we used hard thresholding to remove the noise. The function is calculated as:

$$f\left(C_{s,w,k1,k2,k3}\right)=\left\{\begin{array}{c} C_{s,w,k1,k2,k3},\left|C_{s,w,k1,k2,k3}\right|\geq C\sigma_{\lambda }\sigma \\ 0,\left|C_{s,w,k1,k2,k3}\right|<C\sigma_{\lambda }\sigma \end{array}\right.$$ (2)

where *σ* is the noise variances; $\sigma_{\lambda }$ is the estimation of noise standard deviation; *C* indicates the scale-dependent coefficient for different scales. For stripe noise, it has a single direction, and the intensity is generally higher than the background. The filter is thus designed as:

$$f\left(C_{s,w,k1,k2,k3}\right)=\left\{\begin{array}{c} C_{s,w1,k1,k2,k3},\left|C_{s,w1,k1,k2,k3}\right|<\text{mean}\\ 0,\left|C_{s,w1,k1,k2,k3}\right|>\text{mean} \end{array}\right.$$ (3)

*w1* belongs to $W_{s}$, where $W_{s}$ contains sub-bands that exhibit a direction like noise and mean is the average signal on a sub-band.

### Vessel enhancement of 3D vascular volumes

Subsequently, vessel enhancement was used to separate the blood vessel signals from the background. During artifact removal, we inevitably compromised the quality of the original image, meaning that a trade-off between denoising effect and image quality is required. Thus, some artifacts remain in the denoised volume. Furthermore, because of the smoothing effect of the denoising algorithm, the contrast between the vessel and background decreases, making it difficult to identify vessel boundaries. To construct high-quality 3D vessel volume, we applied OOF for vessel enhancement, which is a novel curvilinear structure detector. The OOF algorithm demonstrates significant advantages in 3D vascular optimization. By calculating the gradient vector field and determining the optimal direction to enhance vascular structures, it accurately identifies vessel edges while minimizing false positives and missed detections, particularly excelling in the extraction of microvascular density. Additionally, the OOF algorithm exhibits minimal influence from surrounding tissues, effectively distinguishing vessels from nonvascular structures and reducing the risk of leakage. Its high degree of automation and rapid processing speed make it well-suited for handling large datasets. Compared with the Frangi filter based on Hessian matrix, an algorithm that is widely used for structure detection, OOF requires less computational resources and exhibits higher robustness against interference from adjacent curvilinear structures. OOF detects curvilinear structures by finding an optimal direction *W* (*r, x*) and minimizing the oriented flux. The oriented flux can be calculated by calculating matrix *Q* (*r, x*). When oriented flux reaches its minimum value, *Q* (*x, r*) is calculated as follows:

$$Q\left(x,\;r\right)=\lambda WW^{-1}$$ (4)

where *λ* is the eigenvalue of Q (*x, r*). In volume images, Q (*x, r*) is a 3 × 3 matrix and there are 3 different pairs of *λ* and *W*. In images where the intensity of vessels is higher than the background, we considered $\lambda_{1}$ ≤ $\lambda_{2}$ ≤ $\lambda_{3}$ ≈ 0. Different combinations of these eigenvalues as the final response value can be used to detect different types of curved structures. In the OCTA volume, vessels are displayed like a curved structure with an elliptical cross-section. Finally, we set the response values for each voxel as follows:

$$R(x)=\text{max}\left\{\underset{2\leq r\leq 6}{\text{max}}\left\{-\frac{1}{r^{2}}\lambda \right\},0\right\}$$ (5)

### Binarization and 3D rendering

To avoid creating rough surfaces, we applied a 5 × 5 × 5 Gaussian kernel function in a convolution to each voxel to generate a smooth image. Subsequently, an appropriate threshold value was selected to generate 3D binary images. The use of a skeleton representation of the image can preserve the topology of the vessels and reduce redundant information. We skeletonized the vessel images to generate skeleton maps before calculating the vessel parameters. This procedure is performed using the image processing package Fiji (2017 version) [52]. The general idea is to iteratively erode the binary images iteratively until only the skeleton remains. As shown in Fig. 4A, we acquired 3D binary images and their skeleton images.

To obtain a better 3D display result, further processing was performed using the medical image analysis software Imaris (7.4.2 version, Oxford Instruments Andor). We added “surfaces” based on the original binary image. As shown in Fig. 4B, this step adds additional lighting effects and more realistic textures to the original image. Besides, “surfaces” calculates every independent part’s volume. By setting an appropriate volume size as threshold value, we can exclude the apparently small volume that does not belong to vessel to get a clear 3D vessel demonstration.

### Extraction of vascular parameters

In addition to presenting impressive 3D reconstruction effects, another notable feature of OCTA is its ability to accurately extract quantitative data to describe the progression of diseases. As mentioned before, the development of AD is accompanied by vascular changes. Inspired by some works, we plan to indirectly describe the progression of AD by extracting quantitative vascular parameters, thereby demonstrating the quantitative capabilities of OCTA [26–29]*.*

Vessel area (VA) is the volume (or square) of the vessel area. It is directly related to vessel growth, including the emergence of new microvascular and variations in vessel thickness. For a 3D volume image, VA is calculated as follows:

$$VA=\sum_{(x,y,z)V}f_{b}(x,\;y,\;z)$$ (6)

where V is a point set of region of interest (ROI) region and $f_{b}$ is the binary of image. Vessel area density (VAD) is the volume fraction of blood vessels in a representative area. In this work, the area is defined as the region of the dermis in which the vessels grow. VAD is then calculated as follows:

$$VAD=\frac{VA}{\sum_{(x,y,z)\;V}f_{d}(x,\;y,\;z)}$$ (7)

where $f_{d}$ is the binary image of dermis. It is acquired based on the mask segmented from the structural volume data. VAD indicates the relative growth rate of the vessels.

Vessel skeleton (VS) equals the total length of vessels. Different with VA, vascular density (VD) has nothing to do with vascular size and is defined as follows:

$$VS=\sum_{(x,y,z)V}f_{s}(x,\;y,\;z)$$ (8)

where $f_{s}$ is the binary image. Vessel skeleton density (VSD) is specialized to evaluate the vessel length without considering the vessel size information by calculating the ratio of the length occupied by the vasculature to the total area in the skeleton map, and it has been reported to be useful in the analyses of retinal diseases such as diabetic retinopathy (DR) and age-related macular degeneration (AMD). VSD is calculated as follows:

$$VSD=\frac{VS}{\sum_{(x,y,z)\;V}f_{d}(x,\;y,\;z)}$$ (9)

## Data Availability

Data underlying the results presented in this paper are not publicly available at this time but may be obtained from the authors upon reasonable request.
